# MRI and Endometrial Cancer After FIGO 2023—What’s New? A Narrative Review

**DOI:** 10.3390/cancers18061005

**Published:** 2026-03-20

**Authors:** Marco Gennarini, Roberta Valerieva Ninkova, Valentina Miceli, Federica Curti, Sandrine Riccardi, Benedetta Gui, Stefania Rizzo, Aradhana M. Venkatesan, Stephanie Nougaret, Lucia Manganaro

**Affiliations:** 1 Department of Experimental Medicine, Sapienza University of Rome, 00161 Rome, Italy; 2 Department of Radiological Sciences, Oncology and Pathology, Sapienza University of Rome, 00161 Rome, Italy; 3 Dipartimento Diagnostica per Immagini e Radioterapia Oncologica, Fondazione Policlinico Universitario A. Gemelli IRCCS, 00168 Rome, Italy; 4 Imaging Institute of Southern Switzerland, Ente Ospedaliero Cantonale (EOC), 6900 Lugano, Switzerland; 5 Faculty of Biomedical Sciences, Università della Svizzera Italiana (USI), 6900 Lugano, Switzerland; 6 Division of Diagnostic Imaging, Department of Abdominal Imaging, The University of Texas MD Anderson Cancer Center, Houston, TX 77030, USA; 7 PINKCC Lab, INSERM, Montpellier Cancer Research Institute, University of Montpellier, 34298 Montpellier, France; 8 Department of Radiology, University of Montpellier, 34298 Montpellier, France

**Keywords:** magnetic resonance imaging, endometrial cancer, diffusion weighted imaging, artificial intelligence, radiomic

## Abstract

Endometrial cancer (EC) is the most common gynaecologic malignancy in developed countries, and its diagnostic and prognostic framework has evolved substantially following the introduction of the 2023 FIGO staging system, which integrates molecular classification with clinicopathologic features. Both histopathologic features, such as lymphovascular space invasion (LVSI) and molecular subtype, including POLE mutation status, mismatch-repair deficiency, and p53-abnormal phenotype, are incorporated into the updated staging system, highlighting the importance of tumour biology in risk stratification. Accordingly, the value and contribution of MRI to patient management must extend beyond macroscopic assessment to support a more biologically driven approach. This narrative review synthesizes recent advances in MRI for EC, highlighting developments that improve diagnostic accuracy and align imaging with the molecular paradigm. Multiparametric MRI remains the reference standard for local staging, while emerging quantitative diffusion techniques provide microstructural biomarkers associated with tumor aggressiveness and prognostic features. The consistency of nodal staging has been enhanced by Node-RADS, a structured reporting system that integrates nodal morphology and configuration, with the goal of improving reproducibility and diagnostic performance over size-based assessment alone. Radiomics and artificial intelligence (AI) represent the most transformative frontier, enabling MRI to infer biological behaviours previously accessible only via histopathologic assessment. Radiomics and deep-learning models have demonstrated high accuracy in predicting LVSI, DMI, nodal metastasis, and molecular subtypes, offering non-invasive biomarkers aligned with FIGO 2023 prognostic categories. Together, these advances position MRI as a quantitatively enriched, biologically relevant tool that supports precision oncology in endometrial cancer.

## Simple Summary

Endometrial cancer is the most common gynecologic cancer in developed countries, and its management is changing due to the introduction of the 2023 FIGO staging system, which places greater emphasis on tumor biology rather than anatomy alone. This review explains why magnetic resonance imaging is being reconsidered in this new context. The authors aim to describe how recent advances in MRI techniques can support more accurate diagnosis, risk assessment, and treatment planning. Beyond showing how far a tumor has spread, modern MRI methods can provide information related to tumor aggressiveness and prognosis through advanced diffusion imaging, standardized lymph node evaluation, and computer-based image analysis. These developments may help bridge the gap between imaging and molecular classification, offering non-invasive tools to guide personalized care and future research in endometrial cancer.

## 1. Introduction

Endometrial cancer (EC) is the most common gynaecologic malignancy in Western countries, and its incidence continues to rise, largely driven by population aging and rising obesity rates. Despite its generally favourable prognosis in early stages, EC includes a heterogeneous group of tumours with distinct biological behaviors, ranging from low-grade endometrioid lesions to highly aggressive non-endometrioid and serous variants [1].

A major conceptual shift in EC classification occurred with the publication of the 2023 FIGO (International Federation of Gynaecology and Obstetrics) staging update. The 2023 FIGO staging system for endometrial cancer introduces a structural redefinition of stage that integrates anatomic extent with histopathologic and, when available, molecular parameters [2]. While the system remains surgically based, it formally incorporates tumor type, grade, and substantial lymphovascular space invasion (LVSI), alongside optional molecular classification derived from The Cancer Genome Atlas (TCGA), reflecting the accumulated evidence that tumor biology may outweigh purely anatomic factors in prognostic stratification [3].

Compared with FIGO 2009, staging is no longer based solely on depth of myometrial invasion but integrates histologic aggressiveness, substantial LVSI, and refined nodal and peritoneal categories. Early-stage disease is now subdivided according to tumor biology, and nodal metastases are distinguished between micro- and macrometastases, increasing prognostic precision [2,4,5,6].

A major innovation is the formal acknowledgment of molecular subtypes. ECs are stratified into POLE-mutated (POLEmut), mismatch repair–deficient (MMRd), no specific molecular profile (NSMP), and p53-abnormal (p53abn) groups, each associated with distinct clinical outcomes [7]. POLEmut tumors show excellent prognosis despite frequently high-grade morphology, whereas p53abn carcinomas display aggressive behavior and poor survival; MMRd and NSMP tumors generally confer intermediate risk [3]. When molecular data are available, stage modifiers (e.g., IAmPOLEmut or IICmp53abn) may result in downstaging or upstaging of early-stage disease, underscoring the shift toward biologically integrated staging [2].

For radiologists, these changes do not diminish the central role of imaging in preoperative staging. Accurate assessment of myometrial invasion, cervical stromal involvement, adnexal extension, nodal metastases, and peritoneal dissemination remains essential for correct stage allocation within the revised system [7]. However, because several newly incorporated determinants—such as substantial LVSI and molecular subtype—are not directly assessable with conventional MRI, the 2023 framework emphasizes the need for imaging to be interpreted within a multidisciplinary, biologically informed context [3].

Historically, MRI has been the cornerstone of preoperative assessment, given its high accuracy in evaluating myometrial invasion, a key prognostic determinant strongly associated with nodal metastases, thereby influencing surgical decision-making [8].

MRI also plays a crucial role in selecting candidates for fertility-sparing treatment (FST). Accurate assessment to exclude myometrial invasion, cervical involvement and to evaluate nodal status is mandatory according to European Society of Gynaecological Oncology (ESGO)—European Society of Human Reproduction and Embryology (ESHRE)—European Society for Gynaecological Endoscopy (ESGE) and European Society of Urogenital Radiology (ESUR) criteria and the National Comprehensive Cancer Network (NCCN) [9,10,11]. In addition, MRI aids in cancer diagnosis when an endometrial biopsy is not feasible and is essential for determining the site of tumour origin, particularly in distinguishing endometrial from endocervical malignancies.

This shift presents both a challenge and an opportunity, driving the advancement of quantitative imaging techniques that have the potential to enhance prognostication and treatment planning. Techniques such as diffusion kurtosis imaging and time-dependent diffusion have shown promise in predicting tumour grade, lymphovascular space invasion (LVSI) and even HER-2 expression, expanding the role MRI beyond anatomic delineation into the realm of functional and microstructural assessment [12].

Parallel advances in radiomics and artificial intelligence now aim to extract imaging biomarkers capable of approximating histopathologic and molecular determinants, further extending MRI beyond purely morphological assessment.

The aim of this review summarizes recent innovations in MRI techniques and applications for EC.

## 2. Search Strategy

A comprehensive literature search was conducted across PubMed, Web of Science, and Scopus. For PubMed, the following search string was applied: (“endometrial cancer” OR “endometrial carcinoma”) AND (“magnetic resonance imaging” OR MRI) AND (“FIGO 2023” OR “molecular classification” OR “radiomics” OR “diffusion-weighted imaging” OR “Node-RADS” OR “artificial intelligence”). Equivalent keyword combinations were adapted to Web of Science and Scopus using database-specific syntax. Two reviewers independently screened and extracted data; disagreements were resolved by consensus with a third reviewer.

English-language publications from 1 January 2015 to 13 February 2026 were considered. The search strategy was complemented by a manual review of reference lists from eligible articles to identify additional relevant studies. As this is a narrative, rather than a systematic review/meta-analysis, article selection was guided by thematic relevance to MRI biomarkers, quantitative imaging and AI-based methods, acknowledging that this approach may introduce a degree of subjectivity.

The initial search yielded 120 articles. Titles, abstracts, and methods sections were screened to remove studies unrelated to the objectives of the review. Editorials, letters, conference abstracts, and papers lacking sufficient detail on MRI methodology, pathology, or imaging biomarkers were excluded. Opinion pieces and anecdotal reports were removed during a secondary screening. Ultimately, 58 studies were retained, which provided comprehensive, clinically meaningful information on multiparametric MRI techniques, quantitative imaging, radiomics, artificial intelligence, and pathology–imaging correlations relevant to EC in the era of FIGO 2023.

**Scheme 1:**
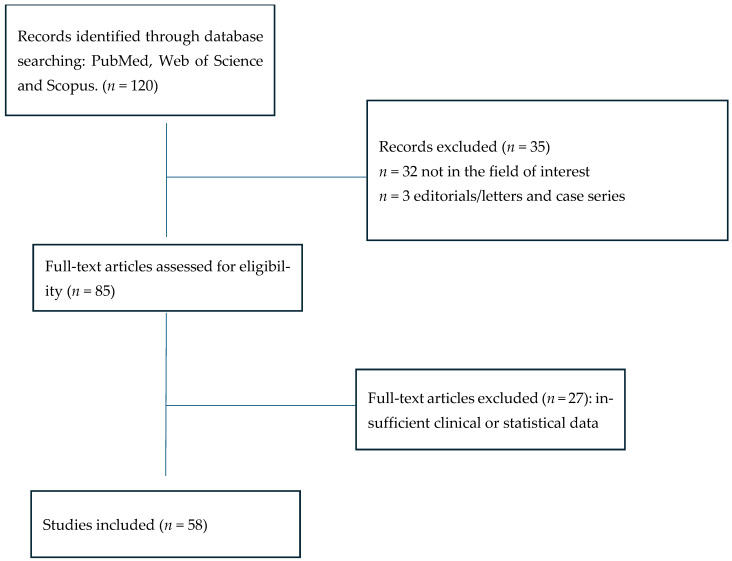
Review flow diagram.

Although this is a narrative review, a structured flow diagram is provided to enhance transparency of study selection (Scheme 1).

To provide a structured overview of the evolving imaging landscape in endometrial cancer and clarify the transition from conventional morphological assessment to quantitative and AI-driven approaches, the main differences are summarized in Table 1.

## 3. State of the Art for MRI Clinical Applications in Endometrial Cancer

### 3.1. Evaluation of Tumor Local Extent

MRI constitutes the most comprehensive and accurate modality for local staging of EC, offering unrivalled anatomical detail and multiparametric functional information compared to other clinical imaging modalities. Its primary clinical application remains the assessment of tumour extent within the uterus—particularly the depth of myometrial invasion—which continues to influence surgical planning and prognostic classification, as per ESUR and ESGO/ESTRO/ESP and the NCCN recommendations. These recommendations derive from international guideline documents and expert consensus statements rather than randomized prospective trials [10,11]. High-resolution T2-weighted imaging enables precise delineation of uterine zonal anatomy, while diffusion-weighted imaging (DWI) improves tumour conspicuity and tumour–myometrial contrast, in line with established multiparametric MRI protocols for EC staging [10,32].

Dynamic contrast-enhanced MRI (DCE-MRI), especially the 35–40 s early post-contrast phase, allows for the assessment of subendometrial enhancement, corresponding to the inner junctional zone, which enhances earlier than background myometrium [32]. Interrupted subendometrial enhancement serves as a reliable surrogate of superficial myometrial infiltration. This has been highlighted in the ESUR-guided imaging framework and validated by recent evidence incorporating updated DCE-MRI performance analysis [33].

**Figure 1:**
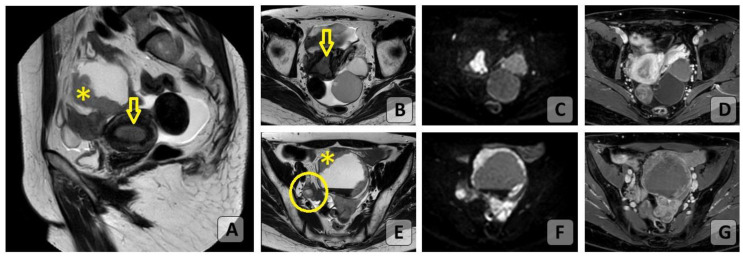
Sagittal T2-weighted (**A**), axial T2-weighted (**B**,**E**), axial DWI (**C**,**F**) and T1-weighted fat-suppressed post-contrast images (**D**,**G**) of a 61-year-old woman with endometrioid carcinoma (arrow) with involvement of the left ovary (*) and right obturator nodal metastasis (circle).

In addition, MRI provides valuable information regarding cervical stromal involvement, parametrial spread, adnexal invasion, and serosal infiltration, findings that can upstage patients and directly influence surgical strategy. As such, MRI imaging findings provide an invaluable road map for the surgeon (Figure 1).

The critical clinical role of MRI lies in the identification of extra-compartmental pelvic disease, including bladder or rectal invasion, vaginal extension and bulky nodal involvement—findings that define stage III–IV disease regardless of molecular profile. MRI’s multiplanar capability allows more accurate assessment of pelvic sidewall and paravaginal region involvement compared to CT, owing to its superior soft-tissue contrast, as demonstrated in comparative staging studies and in structured nodal assessment frameworks such as Node-RADS, which formalized morphology-based criteria for metastatic involvement [34,35].

### 3.2. Limitations of MRI for Risk Stratification in Early Stage Disease

In early-stage disease, MRI plays a critical role in premenopausal patients wishing to preserve fertility. However, its incremental value in early-stage endometrial cancer in postmenopausal patients has changed with the advent of the 2023 FIGO staging system, which recognizes molecular determinants as central prognostic classifiers. Many of these—POLE-mutated status, p53-abnormal phenotype, MMR-deficiency, and LVSI extent—are non-anatomical and therefore invisible on routine MRI [36,37].

In cancers confined to the endometrium or with superficial myometrial invasion, MRI can confirm the absence of gross extrauterine disease but cannot determine molecular risk class, which increasingly dictates recurrence risk and adjuvant therapy. Even LVSI, a key FIGO determinant, cannot be visually assessed. There is data to suggest that LVSI may be assessed indirectly from surrogate imaging markers or advanced quantitative techniques, with some radiomics-based models outperforming conventional MRI in predicting LVSI. However, this remains investigational [38,39].

Thus, while MRI retains an essential supportive role, it is less determinative in low-volume, biologically driven early-stage disease in post-menopausal patients. This represents a central limitation in the contemporary paradigm: MRI excels in mapping macroscopic extension but cannot replace molecular profiling in risk stratification.

Beyond biological invisibility, MRI staging is also limited by technical and interpretative variability. Although high-resolution T2-weighted imaging combined with DWI and DCE improves accuracy for assessing myometrial and cervical stromal invasion [32,33], reported diagnostic performance remains reader-dependent, particularly in cases of adenomyosis, leiomyomas, or post-biopsy changes [9,32]. Interobserver variability has been documented in the evaluation of depth of myometrial invasion and junctional zone disruption, especially in borderline cases, potentially influencing surgical planning [9,36].

Nodal staging represents an additional limitation. Even with structured systems such as Node-RADS [40], MRI primarily relies on morphological criteria, which may fail to detect micrometastases that are identifiable only through ultrastaging of sentinel lymph nodes [41,42,43,44]. The distinction between micrometastases and macrometastases introduced in FIGO 2023 [2,6] further highlights this intrinsic constraint, as current spatial resolution does not allow reliable detection of submillimetric metastatic deposits.

Therefore, while MRI remains indispensable for mapping macroscopic disease extension, its performance in detecting microscopic vascular invasion and nodal micrometastasis remains limited, reinforcing the complementary role of surgical pathology and molecular profiling within the biologically integrated FIGO 2023 staging framework [2,3,7].

### 3.3. Scenario: MRI and Fertility-Sparing Treatment in EC

MRI is central in evaluating eligibility for fertility-sparing treatment (FST) in endometrial cancer. International guidelines (ESGO/ESHRE/ESGE and NCCN) restrict conservative management to grade 1 endometrioid carcinoma strictly confined to the endometrium and require MRI to exclude myometrial and cervical stromal invasion, adnexal disease, and lymphadenopathy prior to treatment. These indications are based on consensus-driven guideline recommendations supported predominantly by observational outcome data [9,10,11]. In addition, candidates for fertility-sparing treatment should have no evidence of Lynch syndrome, given the increased risk of synchronous or metachronous malignancies [9]. This is echoed by the extensive ESUR recommendations, which emphasize MRI as the most accurate tool for assessing local tumour extent, establishing strict imaging criteria and protocol standardization for FST pathways [10,11].

**Figure 2:**
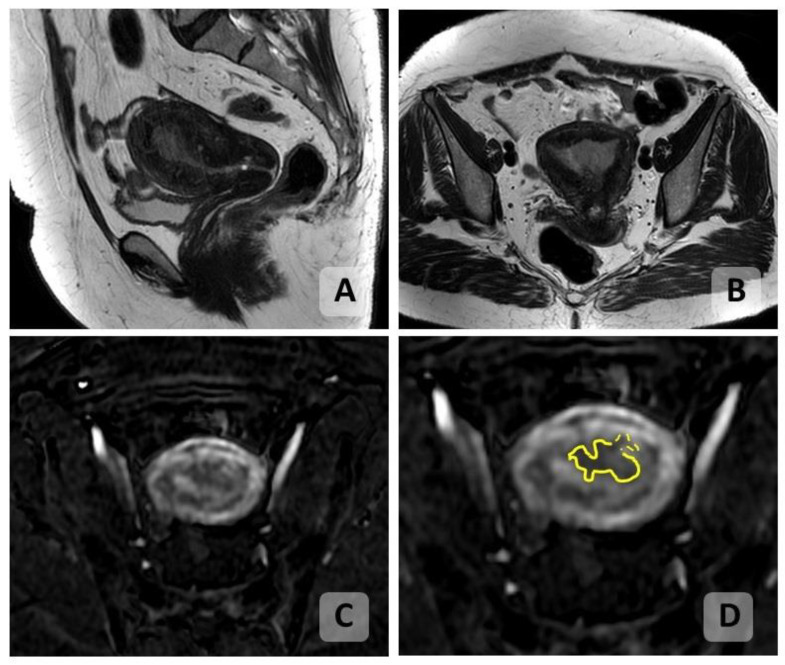
Sagittal T2-weighted (**A**), axial T2-weighted (**B**), and early subtracted T1-weighted images (**C**,**D**) of a 40-year-old patient with endometrioid carcinoma. Figures (**C**,**D**) show early subtracted T1-weighted images demonstrating interruption of the early subendometrial enhancement rim by the tumor, as highlighted by the yellow dashed line in (**D**).

The absence of myometrial invasion represents the key imaging prerequisite, as even minimal infiltration increases the risk of nodal disease and compromises the oncologic safety of conservative management [33]. Within MRI protocols, dynamic contrast-enhanced imaging plays a decisive role: evaluation of early subendometrial enhancement (35–40 s after contrast administration) allows assessment of the integrity of the inner junctional zone. Interruption of this early enhancement rim suggests myometrial invasion and contraindicates fertility preservation [33] (Figure 2).

MRI also supports ongoing clinical decision-making during FST, clarifying discordant biopsy results, monitoring suspected progression, and providing essential follow-up data. Per current ESUR follow-up pathways, MRI is recommended at structured intervals during the first years after diagnosis [10]. Per the NCCN, repeat pelvic MRI is recommended for patients with persistent endometrial carcinoma after 6–9 months of ineffective treatment, particularly if considering further FST, to inform next steps in management [11].

## 4. New Advances

### 4.1. Node Reporting and Data System (Node-RADS)

Accurate assessment of lymph node involvement is a key determinant of prognosis and adjuvant treatment selection in EC [41,42]. Sentinel lymph node (SLN) mapping has progressively replaced systematic lymphadenectomy in most early-stage cases, given its high sensitivity and lower morbidity [41,42,43,44]. Nevertheless, SLN mapping is not universally feasible, and preoperative imaging remains essential for surgical planning and risk stratification.

Traditional MRI evaluation based solely on size criteria shows limited sensitivity, as metastatic nodes may be normal-sized [35,45].

In this context, a standardized and reproducible MRI-based approach to nodal assessment is crucial, providing the rationale for the introduction of structured systems such as Node Reporting and Data System (Node-RADS).

Node-RADS integrates dimensional thresholds with qualitative features—texture, border, and shape—to produce a 5-point likelihood score that reflects the probability of nodal metastasis [40].

Evidence supporting its use in EC is increasingly robust. Liu et al. demonstrated excellent diagnostic performance (AUC 0.93 at patient level; 0.91 at nodal-station level) and near-perfect inter-reader agreement (κ up to 0.94), identifying a cutoff >2 as optimal for metastatic involvement [35].

Bonatti et al. further showed that Node-RADS is more accurate and reproducible on MRI than CT, with MRI achieving 100% specificity in experienced readers [34].

Riccardi et al. expanded its relevance by demonstrating significant correlations not only with histologically confirmed nodal metastasis (AUC 0.832), but also with deep myometrial invasion, aggressive histology, and LVSI—factors integrated into the FIGO 2023 framework [37].

Beyond diagnostic performance, Node-RADS improves standardization and multidisciplinary communication by replacing non-uniform descriptors with a structured probability-based system [45]. Complementary evidence from radiomics studies supports the biological rationale underlying morphology-based nodal assessment: combining ADC values and radiomic features from the primary tumor significantly improves prediction of pelvic nodal metastasis (AUC 0.94), outperforming morphology alone [25].

**Figure 3:**
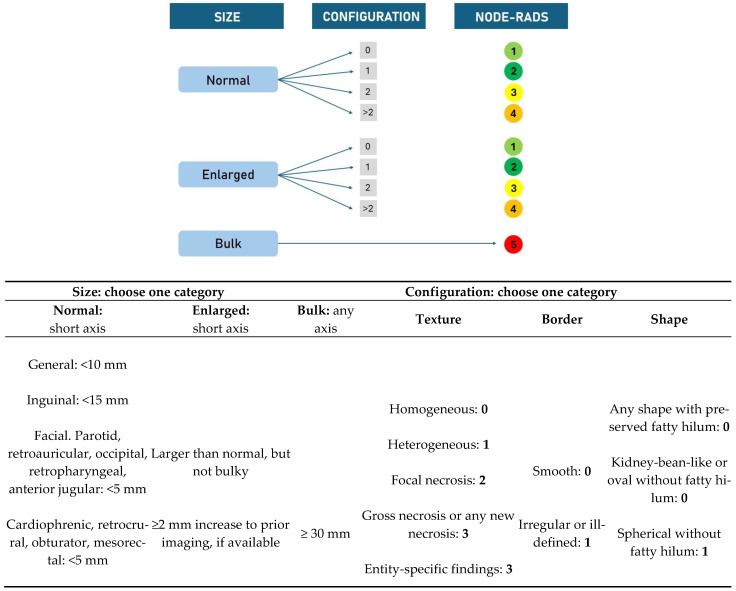
Description of the Node-RADS scoring system flowchart criteria, adapted from the original publication [45].

Collectively, these data support Node-RADS as a reproducible and clinically meaningful MRI-based tool for nodal staging in EC, aligned with contemporary risk-adapted management strategies. Current supporting evidence is derived from retrospective validation studies, and prospective outcome-based validation in gynecologic oncology remains limited [34,35,37,45] (Figure 3).

### 4.2. Diffusion-Weighted Imaging in EC: From Conventional ADC to Advanced Microstructural Models

**Figure 4:**
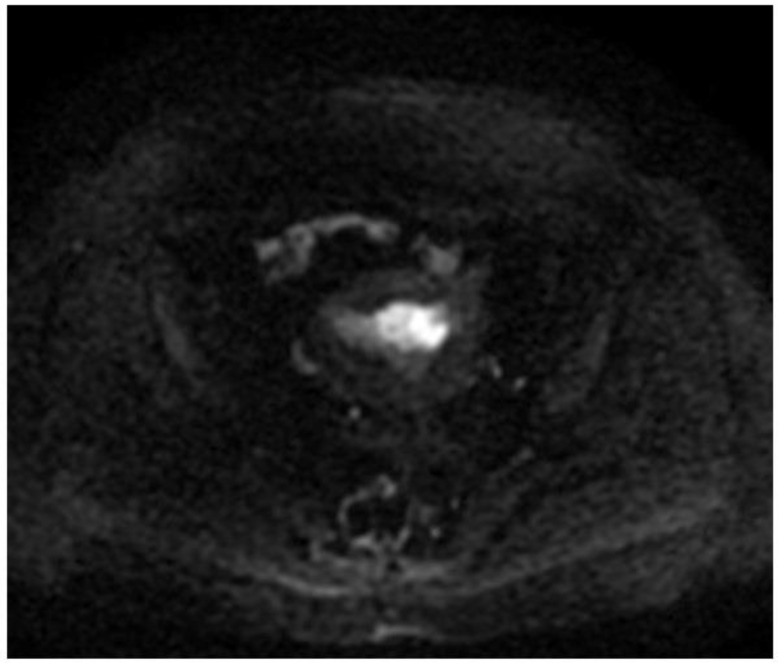
Axial PGSE diffusion-weighted images (b = 1000) of the patient shown in Figure 2.

DWI has become an essential component of MRI assessment in EC, offering quantitative information on tumour cellularity and microstructure that complements morphological imaging. Conventional DWI, based on pulsed-gradient spin-echo (PGSE) sequences, provides the ADC, a parameter that reflects the degree of restricted diffusion (Figure 4).

Diffusion-weighted imaging has been extensively investigated in EC as a non-invasive biomarker of tumour aggressiveness. Early studies primarily focused on the ADC as a surrogate of tumour cellularity, reporting inverse correlations with histological grade, depth of myometrial invasion, and LVSI, thus supporting its potential role in preoperative risk stratification [13,14]. Subsequent investigations evaluated whether ADC could act as an “imaging biopsy”, demonstrating associations with tumour grade, LVSI, and FIGO stage, with heterogeneous and sometimes conflicting results across cohorts [15,16,17]. With the publication of the FIGO 2023 staging system and the growing clinical relevance of molecular classification, research has progressively shifted from purely morphologic and histopathologic correlates toward imaging biomarkers capable of capturing tumour biology. According to the updated ESUR guidelines, integrating DWI with T2-weighted and contrast-enhanced imaging improves the detection of myometrial invasion and the overall accuracy of staging, justifying its inclusion in all multiparametric MRI protocols for EC [33].

A growing body of evidence demonstrates that ADC correlates with several pathological and biological features. Zhang et al. (2024) [18] showed that ADC values vary significantly across molecular subtypes, with p53-abnormal cancers exhibiting higher ADC than POLE-mutated, MMR-deficient, and NSMP subgroups. This was attributed to the preponderance of serous tumors amongst those which are p53-abnormal, suggesting that diffusion metrics may serve as indirect imaging surrogates of underlying molecular phenotype [18].

At the same time, conventional ADC remains a composite marker influenced by multiple microstructural components—cell density, membrane permeability, intracellular-extracellular water exchange, and stromal architecture. Standard PGSE DWI is unable to disentangle the individual contributions of these factors, motivating the development of advanced diffusion models capable of probing tissue microstructure with greater specificity [19].

Among these advanced approaches, diffusion kurtosis imaging (DKI) is one of the most established. By quantifying non-Gaussian water diffusion, DKI captures tissue heterogeneity more accurately than mono-exponential ADC. It has been demonstrated that kurtosis analysis provides a clear delineation of the junctional zone and reliably distinguishes endometrial carcinoma from normal uterine layers, with kurtosis and diffusivity values showing strong correlations with histological grade and effectively differentiating metastatic from non-metastatic lymph nodes [20]. In the study by Maiuro et al. (2025), kurtosis values were significantly elevated in tumour and peritumoral tissue compared with normal endometrium, and diffusion clustering further demonstrated heterogeneous diffusion compartments within cancers, supporting the role of DKI as a tool for virtual microstructural mapping [21].

**Figure 5:**
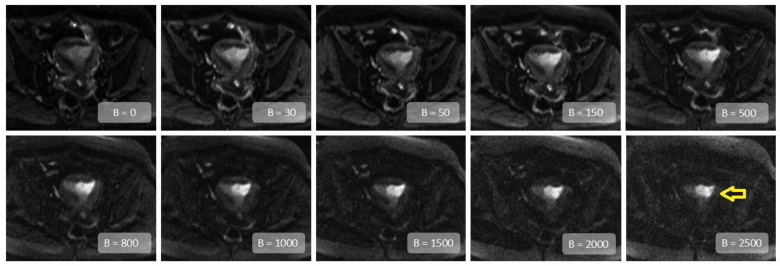
Axial PGSE diffusion-weighted images (b = 1000) of the patient shown in Figure 2. The tumor is indicated by the arrow.

Beyond kurtosis-based metrics, intravoxel incoherent motion (IVIM) imaging further extends diffusion analysis by separating true molecular diffusion from perfusion-related effects, enabling the extraction of quantitative parameters that reflect tumor cellularity and microvascularity and showing promising associations with tumor grade, risk stratification, and treatment response in endometrial cancer [19] (Figure 5).

Time-dependent diffusion MRI (TDD-MRI) represents an additional advancement, leveraging the dependence of diffusion on the effective diffusion time, enabling assessment of microstructure at multiple spatial scales. Two complementary implementations of TDD-MRI are increasingly applied to EC:

1. Oscillating-Gradient DWI (OGSE), which achieves short diffusion times and emphasizes smaller-scale structural restrictions.

The study by Ejima et al. (JMRI 2024) reported that the ADC ratio derived from OGSE and PGSE (ADCOGSE/ADCPGSE) correlates strongly with histological grade, LVSI, FIGO stage, and prognostic risk classes, outperforming either ADC value alone [22].

2. IMPULSED microstructural mapping, which derives quantitative parameters such as cell diameter, intracellular volume fraction (Vin), extracellular diffusivity (Dex), and cellularity.

Yue et al. (KJR 2025) demonstrated that these microstructural parameters reliably differentiate benign from malignant lesions and identify aggressive ECs, with strong correlations (r = 0.77–0.83) between imaging-derived cellularity and histological reference standards [23].

Zhao et al. similarly confirmed that TDD-derived markers (cellularity, ΔADC metrics) can predict risk stratification and Ki-67 proliferation status in endometrioid carcinoma, adding prognostic value beyond conventional ADC [24].

Collectively, findings across these studies indicate that DWI is evolving from a single-parameter technique into a family of quantitative methods capable of providing detailed insights into tumour biology, aggressiveness, and risk classification. By integrating conventional PGSE, OGSE, kurtosis imaging, and microstructural TDD-MRI models, diffusion imaging has become one of the most dynamic and informative components of MRI assessment in EC.

Compared with conventional ADC measurements, advanced diffusion models provide incremental sensitivity to microstructural heterogeneity; however, their additional clinical value over standard DWI requires prospective multicenter validation before routine implementation.

## 5. Radiomics and AI

Radiomics refers to the computational extraction of high-throughput quantitative features from medical images, converting standard radiologic examinations into mineable datasets that characterize tissue heterogeneity, microstructural complexity, and tumor phenotype beyond human visual perception [30,46]. The radiomics workflow typically includes tumor segmentation, feature extraction (shape-based, first-order intensity, texture, and wavelet features), feature selection, and subsequent model construction using machine learning algorithms. These features are intended to capture spatial complexity and biological variability that may reflect tumor aggressiveness and underlying molecular alterations [26]. It should be emphasized that most radiomics and AI investigations in endometrial cancer are retrospective, single-center studies with heterogeneous methodologies, and no imaging-based radiomic biomarker is currently incorporated into formal staging guidelines.

T2-weighted imaging, DWI, and contrast-enhanced sequences provide a suitable substrate for radiomic analysis due to their high soft-tissue contrast and functional information [46,47]. Several studies have demonstrated that MRI-based radiomics models can predict key prognostic and biological factors in EC, including deep myometrial invasion, lymphovascular space invasion, microsatellite instability, POLE mutation status, lymph node metastasis, and recurrence risk [30,31,48].

Beyond traditional “handcrafted” radiomic features, deep learning approaches allow automatic feature learning directly from imaging data through convolutional neural networks, potentially reducing operator dependency and enabling multitask prediction frameworks [48,49]. A recent systematic review and meta-analysis further supports the moderate-to-high diagnostic performance of MRI-based machine learning models for preoperative risk stratification in EC [26].

Although reported diagnostic performances are encouraging, substantial heterogeneity in segmentation strategies, feature selection pipelines, imaging protocols, and validation designs limits direct cross-study comparison and warrants cautious interpretation.

Within this context, radiomics and AI-based approaches represent a methodological extension of MRI, aiming to bridge the gap between macroscopic imaging findings and microscopic tumor biology—an increasingly relevant need in the era of biologically integrated staging systems.

### 5.1. Radiomics and Molecular Subtypes

Several studies have explored the ability of radiomics to noninvasively infer key biological characteristics. One of the most significant contributions comes from the development of multiparametric MRI radiomics models for predicting POLE mutation status, a molecular feature with major prognostic implications. Lin et al. (2023) demonstrated that integrating features from T2-weighted, DWI, and contrast-enhanced T1-weighted images produced a radiomics model (RM2) with excellent discriminatory ability, achieving AUCs of 0.885 and 0.810 in training and validation cohorts, respectively, thus offering a feasible imaging surrogate for identifying POLE-ultramutated tumours [31].

This is particularly relevant given the cost and limited availability of sequencing platforms in clinical practice. Beyond identifying POLE-mutated tumors, radiomics has also proven effective for predicting a broader range of molecular and pathological features. In the study by Ma et al. (2023) [30], a large cohort of 292 patients was used to evaluate radiomics models for predicting MSI status, LVSI, DMI, and HER-2 expression. Random forest classifiers achieved AUCs of 0.844 for MSI, 0.952 for LVSI, and 0.840 for DMI, while combined clinical–radiomic models further improved predictive performance in most subsets, providing promising tools for preoperative risk stratification and for anticipating molecular signatures that guide adjuvant therapy [30].

### 5.2. Radiomics/AI and LVSI

Substantial LVSI is one of the strongest adverse prognostic factors in endometrial cancer, independently associated with pelvic recurrence and distant metastasis (HR up to 6.2) [50].

Despite its clinical relevance, LVSI remains assessable only on hysterectomy specimens. Conventional MRI features alone provide limited predictive capability; for example, tumor short-axis diameter demonstrated modest performance (AUC 0.61), confirming that macroscopic morphology cannot reliably capture LVSI biology [51].

Radiomics and machine learning approaches aim to bridge this gap by extracting quantitative features reflecting microstructural heterogeneity and vascular alterations. In external validation, T2-weighted radiomics models have achieved AUC values around 0.85 for LVSI prediction, outperforming visual assessment [38].

A meta-analysis pooling nine MRI-based LVSI studies reported a summary AUC of 0.82, with radiomics models consistently exceeding morphology-only approaches [39]. More recent multiparametric models integrating T2-weighted, diffusion-weighted, and contrast-enhanced sequences have reported AUC values approaching 0.90–0.91 [27,28], suggesting incremental benefit from combining diffusion and perfusion-derived features. Reproducibility has been addressed in IBSI-compliant pipelines with external validation cohorts, demonstrating stable performance (AUC ≈0.80) and improved methodological robustness [29].

Advanced diffusion techniques further support the biological plausibility of imaging-based LVSI prediction, as non-Gaussian diffusion metrics and time-dependent diffusion parameters correlate with tumor aggressiveness and microstructural complexity [21,22,23,24].

Overall, MRI-based LVSI prediction represents one of the most consistently investigated applications of radiomics in endometrial cancer, with reproducible moderate-to-high diagnostic performance. However, most studies remain retrospective, and broader multicenter validation is required before routine clinical implementation.

### 5.3. Radiomics/AI and Prognosis

The ability of radiomics to capture patterns associated with nodal metastasis and recurrence has also been investigated. Lin et al. (2024) [46] developed a DWI-based radiomics risk score (Rad-Score) capable of predicting high-risk EC associated with nodal metastasis or recurrence. Their model achieved accuracy rates of 71.1% and 71.0% in training and test cohorts, respectively, and correlated strongly with elevated choline metabolites on MR spectroscopy, linking radiomic signatures to metabolic reprogramming in aggressive tumours [46].

This integration of imaging phenotypes with metabolic biomarkers highlights one of the key strengths of radiomics: the ability to noninvasively interrogate tumour biology from multiple complementary angles. Song et al. (2025) [47] applied consensus clustering to identify MRI radiomics phenotypes in intermediate-to-high-risk EC, revealing two phenotypes with markedly different associations to DMI, cervical mucosal infiltration, and recurrence risk. Their combined radiomics–FIGO staging model improved prognostic stratification, with concordance indices rising from 0.66 (radiomics alone) to 0.72 when combined with staging [47].

These data confirm that radiomics is capable not only of predicting individual histopathological markers, but also of defining imaging-based tumour subtypes that parallel biologically meaningful categories.

Radiomics also finds significant application in scenarios where conventional MRI interpretation is challenging or infeasible. Jiang et al. (2023) [52] developed radiomics models specifically designed for MRI-invisible early-stage EC—lesions that escape typical radiological detection. Using features extracted from sagittal T2-weighted and contrast-enhanced T1-weighted images, they achieved AUCs of 0.873–0.918 for detecting MRI-invisible EC and AUCs of 0.834–0.854 for predicting myometrial invasion, demonstrating that quantitative imaging features can reveal diagnostic signals inapparent to the human eye [52].

This finding is particularly valuable in the context of FST, for which early-stage invisible lesions must be accurately identified in order to inform appropriate management.

DL has further expanded the horizon of MRI-based intelligence in EC. Chen et al. (2025) [49] developed a multimodal deep learning radiomics (MDLR) model incorporating T2WI and multi-scale DL features extracted via ResNet18. The MDLR achieved high AUCs across multiple validation cohorts (0.862–0.899), significantly outperforming both clinical models and DL-only signatures. The integration of clinical variables with DL image descriptors reflects a trend toward hybrid modelling, where multimodal data fusion yields superior diagnostic performance, particularly for predicting myometrial invasion—a parameter central to both surgical planning and fertility-sparing decision-making [49]. According to Yuan et al., radiomic features combined with clinical variables can identify suitable candidates for conservative treatment, enabling precise patient selection in young women seeking fertility preservation. This integrated approach demonstrated strong performance in predicting 6-month remission after progestin therapy, with AUC values exceeding 0.90 in combined models. These findings confirm MRI’s evolving role, from a purely morphological staging tool to a platform for advanced computational modeling and personalized prediction [53].

DL applications extend beyond myometrial assessment to predicting lymph node metastasis (LNM) and LVSI. In the work of Wang et al. (2024) [48], a multi-task DL model was trained to simultaneously segment endometrial lesions using nnU-Net and predict LNM and LVSI directly from MRI inputs (T2WI, DWI, Contrast-Enhanced T1WI). Their system achieved AUCs up to 0.895 for LNM and 0.848 for LVSI in training cohorts, outperforming radiologists in external testing. This supports the hypothesis that DL can uncover subtle imaging patterns associated with microscopic vascular and nodal invasion that evade conventional interpretation, providing valuable preoperative predictors for surgical staging decisions [48].

The cumulative evidence summarized in a recent systematic review and meta-analysis by Gao et al. (2025) reported pooled radiomics performance metrics across multiple studies, with a pooled sensitivity and specificity of 0.85/0.82 for predicting high-grade EC, 0.80/0.85 for DMI, 0.85/0.73 for LVSI, 0.79/0.85 for MSI, and 0.90/0.72 for LNM [26]. These findings confirm the robust diagnostic potential of MRI-based ML models and highlight sources of heterogeneity such as segmentation protocols and model variability—key considerations for future standardisation.

Together, these studies establish radiomics and AI as central components of modern MRI evaluation in EC. They offer noninvasive alternatives to histopathology for assessing molecular features, robust predictors of invasion and metastasis, and refined tools for prognostic stratification. By integrating DL, radiomic clustering, metabolic correlations, and multimodal fusion, MRI has transitioned into a computational imaging platform capable of addressing the diagnostic challenges posed by tumour heterogeneity and the updated FIGO 2023 staging system.

A major limitation of these studies is the lack of explicit reporting of adherence to IBSI standards, which may hinder reproducibility and comparability of radiomic features across studies (Table 2).

## 6. Future Perspectives

Future developments in MRI assessment of EC are likely to arise from the convergence of multiparametric imaging, quantitative biomarkers, and artificial intelligence. The progression from conventional morphological analysis toward diffusion-based microstructural modelling—such as diffusion kurtosis, OGSE diffusion, and time-dependent diffusion MRI—offers a pathway to probe tumour architecture at scales previously inaccessible to standard PGSE-based DWI, allowing more detailed characterization of cellularity, microvascularity, and stromal organization as demonstrated in the studies by Maiuro et al., Ejima et al., Yue et al., and Zhao et al. [21,22,23,24].

Radiomics and ML will likely accelerate this transition; Li et al. and Meng et al. have shown that radiomic signatures are capable of predicting LVSI, molecular subtypes, and nodal involvement with substantially better accuracy than conventional interpretation [38,39].

DL models—such as the multimodal architectures described by Chen et al. and Wang et al.—afford the possibility of automated lesion segmentation, predictive modelling, and simultaneous multi-task classification of myometrial invasion, LVSI, and nodal metastasis, directly from raw MRI data [48,49].

At the same time, nodal staging stands to benefit from the integration of Node-RADS with radiomic descriptors and clinico-molecular features, as described in the commentary by Méndez et al. and supported by the correlations observed in Riccardi et al. and Liu et al. [35,37,45]. Ultimately, future imaging strategies may rely on algorithmically integrated platforms where MRI, radiomics, and AI converge into unified predictive models tailored to FIGO 2023 molecular categories.

The 2023 FIGO revision has practical implications for MRI reporting. Although assessment of myometrial invasion remains essential, depth of invasion alone no longer determines early-stage allocation, as biologic factors are now integrated into staging [2,3]. Radiologists should therefore continue to quantify the percentage of myometrial involvement and clearly distinguish superficial from deep invasion, in accordance with established MRI staging recommendations [32], while recognizing that final stage assignment may be modified by histologic and molecular determinants [2,6].

Careful evaluation of cervical stromal invasion remains critical, as true stromal extension continues to upstage disease and directly influences surgical management [6,7]. Similarly, nodal assessment has gained additional relevance with the distinction between micrometastases and macrometastases (IIIC1i/ii; IIIC2i/ii) introduced in FIGO 2023 [2,6], reinforcing the need for structured morphology-based evaluation and standardized reporting systems such as Node-RADS [34,35,40].

Importantly, substantial LVSI and molecular classification—now formally incorporated into staging—are not directly assessable by conventional MRI [2,3,7], highlighting the intrinsic limitation of imaging in biologically integrated staging and supporting the investigation of quantitative and radiomics-based imaging biomarkers as potential noninvasive surrogates [26,30,38].

### Large Language Models

The growing adoption of structured MRI reporting represents a critical enabler for the next generation of AI-driven imaging workflows in EC. Structured, anatomy- and feature-based reports improve consistency, completeness, and adherence to clinical guidelines, while simultaneously generating machine-readable data that can be leveraged for advanced computational analysis [54,55]. Recent studies across multiple oncologic imaging domains have demonstrated that large language models can reliably transform free-text radiology reports into structured formats, extract key imaging features, and support standardized staging and categorization systems, including RADS-based frameworks and TNM assignment, with high accuracy [56,57,58]. Although these applications are not yet specific to EC, they provide a robust proof of concept for future FIGO-aligned MRI reporting, enabling automated report synthesis, quality control, guideline-compliance checks, and decision support.

Beyond text-level automation, emerging agentic AI systems represent a further conceptual shift. Unlike task-specific models, agentic AI frameworks are designed to reason across complementary data sources, dynamically integrating MRI-derived imaging features with histopathological and molecular data. By operating across these modalities, such systems may provide deeper insight into tumour biology, refine prognostic stratification, and support individualized risk assessment within the context of the 2023 FIGO molecular classification. These approaches move beyond single-modality prediction toward holistic, patient-centric decision support, positioning MRI as an integral component of integrated imaging–pathology–molecular workflows.

## 7. Conclusions

The 2023 FIGO revision has shifted endometrial cancer staging toward a biologically integrated framework, reinforcing the central role of MRI in preoperative assessment. Beyond conventional anatomical evaluation, structured nodal scoring systems, advanced diffusion techniques, and radiomics-based models expand MRI toward noninvasive estimation of LVSI, molecular risk, and recurrence potential. However, heterogeneity in methodologies, limited multicenter validation, and the need for standardized acquisition and modeling pipelines currently limit routine implementation. Future research should focus on harmonization and prospective validation to integrate quantitative MRI and AI tools within established clinical guidelines.

## Figures and Tables

**Table 1:** Conceptual comparison between conventional MRI assessment and emerging quantitative and AI-based approaches in endometrial cancer.

Domain	Conventional MRI Approach	Structured/Quantitative MRI	Radiomics/AI-Based Models	Clinical Implications in the FIGO 2023 Era
Local Tumor Staging	Visual assessment of myometrial invasion on T2WI and DCE	Quantitative ADC thresholds; advanced diffusion models (DKI, IVIM, OGSE, TDD) [13,14,15,16,17,18,19,20,21,22,23,24]	ML/DL models predicting DMI, grade, MSI, POLE	Supports biologically integrated risk stratification
Nodal Staging	Size-based criteria (short-axis diameter)	Node-RADS structured scoring integrating morphology + size [25,26]	Radiomics-based prediction of LNM from primary tumor and nodal features	Refined nodal risk estimation aligned with micrometastasis-aware staging
LVSI Assessment	Not directly assessable	Indirect diffusion/perfusion indicators	Radiomics models (AUC ~0.80–0.91); DL multitask models [27,28,29]	Potential preoperative surrogate of microscopic vascular invasion
Molecular Subtype Prediction	Not assessable by morphology	Diffusion heterogeneity patterns	Radiomics prediction of MSI, POLE, p53-abn [30,31]	Imaging-based approximation of molecular risk groups
Reproducibility	Reader-dependent	Semi-quantitative metrics	IBSI-compliant pipelines; external validation emerging [29]	Toward standardized, reproducible biological imaging
Clinical Paradigm	Anatomical staging	Microstructural characterization	Predictive and biologically oriented modeling	Transition from morphologic to biologically integrated staging

**Table 2:** Table reporting the radiomics studies included in this section and their adherence to IBSI (IBSI = Image Biomarker Standardisation Initiative) standards.

Author	Title	IBSI Status
Lin Y et al. (2024) [46]	Endometrial cancer risk stratification using MRI radiomics: corroborating with choline metabolism	IBSI applicable—compliance not reported (IBSI-unclear)
Song J et al. (2025) [47]	Multi-modality MRI radiomics phenotypes in intermediate-high risk endometrial cancer: correlations with histopathology and prognosis	IBSI applicable—compliance not reported
Jiang X et al. (2023) [52]	Preoperative assessment of MRI-invisible early-stage endometrial cancer with MRI-based radiomics analysis	IBSI applicable—compliance not reported (IBSI-unclear
Chen Y et al. (2025) [49]	Preoperative discrimination of absence or presence of myometrial invasion in endometrial cancer with an MRI-based multimodal deep learning radiomics model	IBSI not applicable
Yuan W et al. (2025) [53]	Using machine learning to predict the prognosis in endometrial cancer patients undergoing fertility-sparing treatment	IBSI applicable—compliance not reported (IBSI-unclear)
Wang Y et al. (2024) [48]	Fully automated identification of lymph node metastases and lymphovascular invasion in endometrial cancer from multi-parametric MRI by deep learning	IBSI not applicable
Gao Y et al. (2025) [26]	Preoperative risk assessment of invasive endometrial cancer using MRI-based radiomics: a systematic review and meta-analysis	Systematic review and meta-analysis; IBSI not applicable

## Data Availability

No new data were created or analyzed in this study. Data sharing is not applicable to this article.

## Abbreviations

The following abbreviations are used in this manuscript:

ADC	Apparent Diffusion Coefficient
AI	Artificial Intelligence
DCE	Dynamic Contrast-Enhanced
DL	Deep Learning
DKI	Diffusion Kurtosis Imaging
DMI	Deep Myometrial Invasion
DWI	Diffusion Weighted Imaging
EC	Endometrial Cancer
ESGE	European Society for Gynaecological Endoscopy
ESHRE	European Society of Human Reproduction and Embryology
ESGO	European Society of Gynaecological Oncology
ESUR	European Society of Urogenital Radiology
FIGO	International Federation of Gynaecology and Obstetrics
FST	Fertility-Sparing Treatment
IBSI	Image Biomarker Standardisation Initiative
IVIM	Intravoxel Incoherent Motion
LNM	Lymph Node Metastasis
LVSI	Lymphovascular Space Invasion
ML	Machine Learning
MRI	Magnetic Resonance Imaging
MDLR	Multimodal Deep Learning Radiomics
MMR	Mismatch Repair
NCCN	National Comprehensive Cancer Network
OGSE	Oscillating-Gradient DWI
Node-RADS	Node Reporting and Data System
NSMP	No Specific Molecular Profile
PGSE	Pulsed-Gradient Spin-Echo
SLN	Sentinel Lymph Node
TCGA	The Cancer Genome Atlas
TDD	Time-Dependent Diffusion
